# Emotional Intelligence-Based Interventions in Individuals with ADHD: Systematic Review

**DOI:** 10.3390/children13040557

**Published:** 2026-04-16

**Authors:** Sandro Gabrieli, Faustino Andrés-Pérez, Lluna Maria Bru-Luna, Manuel Martí-Vilar

**Affiliations:** 1 Department of Basic Psychology, Faculty of Psychology and Speech Therapy, Universitat de València, 46010 Valencia, Spain; gabrieli@alumni.uv.es (S.G.); fausanpe@alumni.uv.es (F.A.-P.); 2 Department of Education, Faculty of Social Sciences, Universidad Europea de Valencia, 46010 Valencia, Spain; lluna.bru@universidadeuropea.es

**Keywords:** ADHD, emotional intelligence, emotional regulation, executive functions, social-emotional adaptation, systematic review

## Abstract

Attention deficit hyperactivity disorder (ADHD) is a neurodevelopmental disorder characterized by inattention, hyperactivity, and impulsivity, compounded by difficulties in emotional regulation, which have sparked growing interest due to their relationship with emotional intelligence (EI). Background/Objectives: The objective of this study was to analyze the effectiveness and characteristics of interventions aimed at developing EI in people diagnosed with ADHD. Methods: A systematic review was conducted following PRISMA 2020 in the Web of Science, Scopus, PubMed, Dialnet, ERIC, and SpringerLink databases. After applying inclusion and exclusion criteria and evaluating methodological quality, 31 studies were selected. Results: The evidence shows that children and adolescents with ADHD have lower levels of EI than the typically developing population, especially in emotional regulation, stress management, adaptability, and interpersonal skills. Interventions focused on emotional training have demonstrated improvements in emotional competencies, self-control, ADHD symptoms, and social functioning. However, variations are observed according to age, clinical subtype, the presence of comorbidities, and the type of informant, as well as heterogeneity in the assessment instruments used. Conclusions: Strengthening EI emerges as a promising complementary strategy for improving the emotional and social adaptation of people with ADHD. It is recommended to move toward longitudinal studies and more personalized interventions tailored to the clinical and developmental characteristics of the disorder.

## 1. Introduction

Attention deficit/hyperactivity disorder (ADHD) is defined by the American Psychiatric Association as a neurodevelopmental disorder characterized by a persistent pattern of inattention, hyperactivity, and impulsivity that significantly interferes with a person’s functioning and development [1]. These difficulties affect not only the individual, but also their family, social, and educational environments. In adolescents, ADHD is associated with a higher probability of academic failure, behavioral problems, and conflicts in family and social interactions, increasing the risk of emotional and social maladjustment in the medium and long term [2].

The estimated global prevalence of ADHD is between 5% and 7% in children and adolescents, and between 2% and 5% in the adult population [3], establishing itself as the most common neuropsychiatric disorder in childhood [4]. However, the literature shows considerable variability between studies, attributable to methodological, cultural, and diagnostic factors, highlighting the complexity of the disorder and the need for integrative approaches [5].

From a clinical point of view, the DSM-5-TR establishes three core symptoms (inattention, hyperactivity, and impulsivity) and distinguishes three presentations: predominantly inattentive, predominantly hyperactive/impulsive, and combined [1]. The heterogeneity among people with ADHD is reflected in the variability of clinical profiles, developmental trajectories, and neurobiological mechanisms [6]. Among the most important predictors of the persistence of the disorder is symptomatic severity, which is linked to poorer functional outcomes in adulthood [7]. Likewise, family variables such as parental educational level or the presence of psychopathology significantly influence its evolution. From a neurobiological perspective, dysfunctions in the dopaminergic and prefrontal systems have been described that affect self-control, planning, and emotional regulation [8].

In recent decades, there has been a notable increase in the frequency of diagnosis, which has driven scientific interest in better understanding the etiology of ADHD, its clinical expression, and the most effective interventions for addressing it, both pharmacological and psychosocial [9]. Currently, there is no curative intervention; therefore, treatments are aimed at reducing symptoms and improving overall functioning [10,11]. Multimodal treatment (integrating pharmacotherapy, psychological interventions, and psychopedagogical support) is the most effective approach in children and adolescents [12]. At the pharmacological level, stimulants such as methylphenidate and lisdexamfetamine, as well as non-stimulants such as atomoxetine, have demonstrated symptomatic efficacy, although they have limitations related to tolerability and adverse effects [13]. These limitations have led to the development of complementary treatments, including cognitive behavioral therapy, parental training, psychopedagogical intervention, and innovative approaches such as neurofeedback and music therapy [14,15].

One of the most relevant functional aspects of ADHD is difficulty with emotional self-regulation. Limitations in this area contribute significantly to the problems of emotional impulsivity and functional impairment observed in many patients [2]. In this regard, it has been suggested that emotional training may be particularly useful as a complement to other therapeutic approaches.

Within these approaches, interventions based on emotional intelligence (EI) have become particularly relevant. EI is defined as the ability to identify, understand, and effectively manage one’s own and others’ emotions, promoting personal and social adaptation [16]. This competence has been linked to psychological well-being, the quality of interpersonal relationships, and academic and work performance [17]. From a conceptual point of view, it can be approached both as a personality trait and from a skill-based approach focused on emotional processing [18]. In addition, it encompasses competencies such as emotion management and control, self-motivation, emotional recognition in others, and relationship management [19].

Given that one of the core features of ADHD is difficulty in adaptively modulating emotional responses, EI-focused interventions emerge as promising tools by offering strategies for more effective emotion management, greater self-regulation, and strengthened self-awareness [20]. These interventions can also help reduce impulsivity and the intensity of emotional dysregulation [8].

Based on this evidence, the main objective of this study is to systematically compile and analyze the available information on EI-focused interventions for people with ADHD, as well as to explore their characteristics in order to identify those that are most effective. The aim is to gain a deeper understanding of the specific emotional needs of this population and to provide scientific evidence to support the integration of EI as a key component in the therapeutic approach to ADHD.

## 2. Materials and Methods

### 2.1. Design

The purpose of this narrative synthesis is to identify, analyze, and classify the available scientific literature on interventions aimed at developing EI in people with ADHD. This approach provides an up-to-date overview of the tools designed to strengthen EI in this population, thereby promoting more effective coexistence and adaptation in daily life. The results also contribute to establishing a solid foundation for future research in this field.

To ensure a rigorous and transparent process, the review was conducted following the criteria established by the PRISMA 2020 statement [21]. The PRISMA 2020 checklist is provided in Appendix A. Similarly, the study protocol was registered in PROSPERO (ID:CRD420251047790; https://www.crd.york.ac.uk/PROSPERO/view/CRD420251047790, accessed on 12 April 2026), thus ensuring its transparency, reproducibility, and methodological traceability.

The literature search was conducted in the SpringerLink, Scopus, Web of Science, Dialnet, ERIC, and PubMed databases, using specific terms related to the study variables and applying Boolean operators tailored to the research objective. Subsequently, a screening process was carried out based on previously established inclusion and exclusion criteria, selecting only those studies that met all of them. To assess the methodological quality of the selected articles, the Newcastle–Ottawa Scale (NOS) [22] was used for observational studies, RoB-2 [23] for randomized controlled trials and ROBINS-I [24] for non-randomized studies, which allowed the identification of strengths, limitations and possible biases in the available literature.

### 2.2. Sources of Information

This review integrated various databases in order to compile a comprehensive and representative set of studies on interventions aimed at developing EI in populations with ADHD. To ensure a global and diverse overview, publications from different geographical and linguistic contexts were included, provided they were written in English or Spanish.

The search equation used specific terms related to ADHD and emotional intelligence, such as: “ADHD,” “Attention Deficit Hyperactivity Disorder,” “emotional intelligence,” “social emotional learning,” “EQ,” “intervention,” “program,” “treatment,” and “therapy.” These terms were combined using the Boolean operators AND, OR, and NOT in order to increase the accuracy and relevance of the results and to exclude studies that were not aligned with the purpose of the research.

At the same time, the PICOS strategy was used to clearly define the focus of the review and formulate the central question of the study:

What strategies and programs have been developed to improve emotional intelligence in the population with ADHD?

Table 1 shows the relationship between the variables of interest and the dimensions of the PICOS strategy, specifying the criteria applied to each category to guide the selection of studies:

Table 2 details the equations used in each database and the number of results obtained. The search process was carried out during February 2026:

The search strategies described above included the Boolean operator NOT in conjunction with the construct “emotional regulation” to conceptually delimit the scope of the review. This decision was made to avoid the mass collection of studies focused exclusively on isolated neurobiological processes or specific mechanisms of emotional regulation that did not explicitly fall within the multidimensional concept of emotional intelligence (EI).

However, it is important to note that emotional regulation is a central component of EI, as demonstrated by various theoretical and empirical models. Therefore, excluding this construct could have led to the omission of key studies that address EI indirectly through emotional regulation.

This also introduces a potential limitation regarding the comprehensiveness of the collected evidence, as some interventions targeting emotional competencies may not have been selected if they were not labeled under EI frameworks. Therefore, although this strategy enhanced the specificity of the search and ensured a clearer conceptual focus on EI-centered interventions, it may also have reduced sensitivity, a factor to consider when interpreting the results.

### 2.3. Selection Criteria

To ensure the quality, relevance, and appropriateness of the studies included, clearly defined inclusion and exclusion criteria were established. These are presented in Table 3:

In addition, only studies with open access to the full text were included. This criterion was established to ensure full access to the methodological details and results necessary for a rigorous evaluation and accurate data extraction. This decision supports the transparency and feasibility of the review process.

## 3. Results

After conducting an exhaustive search of the various selected databases, 2336 publications were identified. From this initial set, 719 records were eliminated due to duplication. Subsequently, during the first screening process (focused on the analysis of titles, abstracts, protocols, book chapters, opinion articles, and conference proceedings), 1455 studies were excluded for not meeting the objectives of the review.

**Figure 1:**
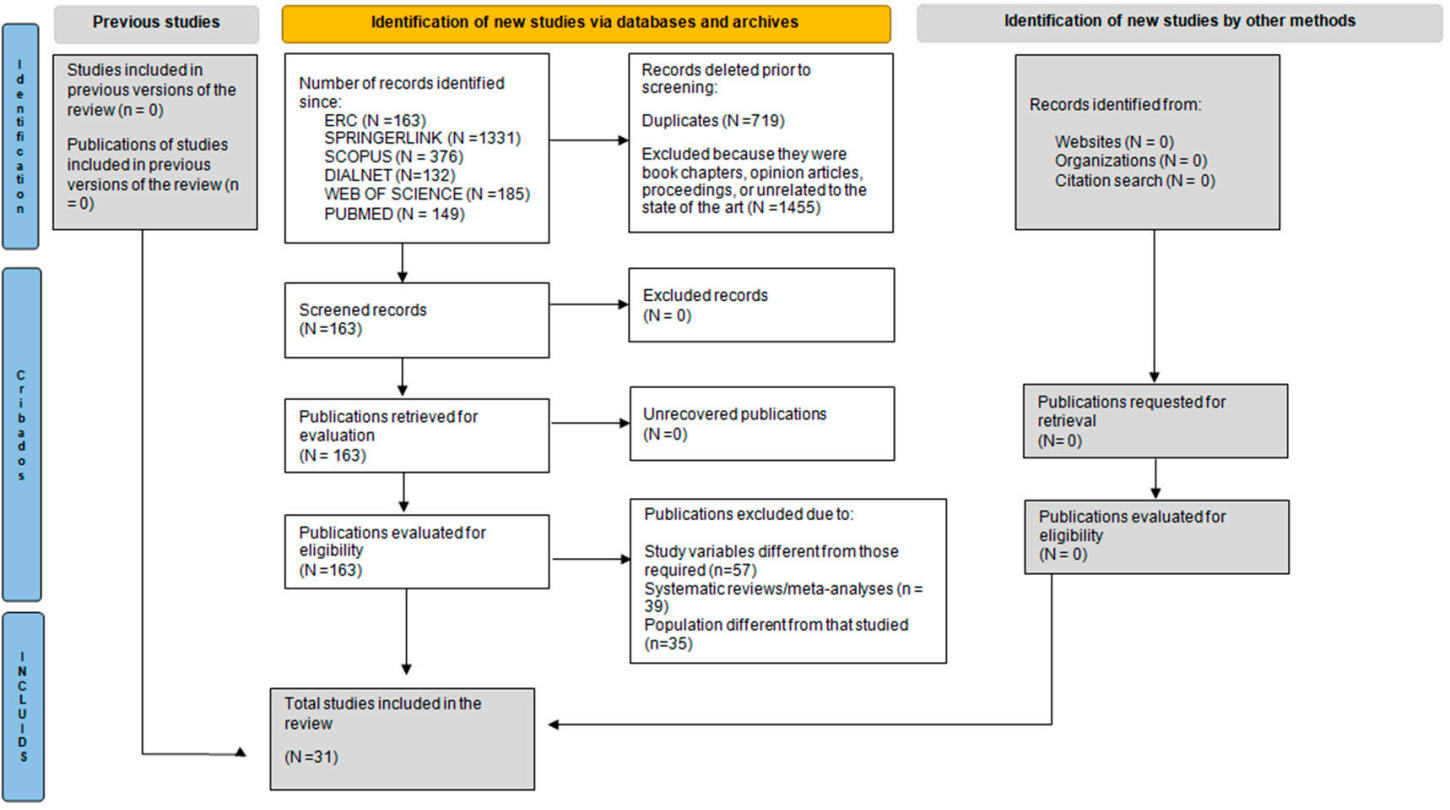
PRISMA flow.

The remaining 163 publications were examined in full text according to the previously established inclusion and exclusion criteria. Finally, 31 articles met all methodological requirements and were included in the narrative synthesis. The entire selection process was carried out rigorously, following a systematic procedure agreed upon by the authors (Figure 1).

The selection process was carried out independently by four reviewers with extensive experience in research methodology and systematic reviews. Each phase was carried out in parallel and blinded between the authors. When there were discrepancies regarding the eligibility of some studies, they were resolved through structured discussion meetings until a unanimous consensus was reached. This process ensured methodological rigor and reduced potential selection bias, guaranteeing the transparency of the process.

### 3.1. Article Quality Assessment Process

The studies included in this review show considerable methodological heterogeneity, incorporating randomized controlled trials, quasi-experimental studies, and cross-sectional and longitudinal observational studies. Therefore, it was not methodologically appropriate or effective to use a single bias risk assessment tool for all articles.

In accordance with international methodological recommendations based on previous systematic reviews, different instruments were used depending on the design of each study, ensuring a rigorous and valid assessment that was tailored to the methodological characteristics of each type of research. Consequently:

- Randomized controlled trials: RoB 2. This instrument assesses the quality of articles by focusing on five domains: (D1) randomization process (sequence generation and concealment), (D2) deviations from planned interventions (adherence and knowledge of allocation), (D3) incomplete outcome data (losses and dropouts), (D4) outcome measurement (blinding and validity of instruments), and (D5) selection of reported outcomes (selectivity in reporting outcomes). Each domain is grouped into low risk, some concerns, or high risk. Subsequently, an overall judgment of methodological quality is made. Table 4 represents the quality assessment of these studies [23].

- Intervention studies, quasi-experimental, non-randomized: ROBINS-I. This tool analyzes studies by looking at seven factors: confounding (C1), participant selection (C2), classification of the intended intervention (C3), deviations from the intended intervention (C4), missing data (C5), measurement of outcomes (C6), selection of reported outcomes (C7). Overall bias is grouped into low, moderate, serious, or critical. Table 5 shows the quality assessment of these interventions [24].

- Observational studies: Newcastle–Ottawa Scale (NOS). This tool analyzes three fundamental dimensions, establishing a maximum score of 10 points [22].
  - Item 1: Selection (0 to 5 points): Representativeness and sample size, adequate definition and categorization of groups, and validity in measuring exposure.
  - Item 2: Comparability (0 to 2 points): Control of confounding variables and statistical adjustment between groups.
  - Item 3: Results (0 to 3 points): Validity and reliability of measurement instruments, adequacy of statistical analysis, and clarity in the presentation of findings.

To group the studies according to their quality level, the following classification was established: high- (8–10 points, low risk of bias), medium- (6–7 points, moderate risk), and low-quality (≤5 points, high risk of bias). Table 6 shows the quality assessment of the articles according to this instrument.

This decision allowed for a differentiated, consistent, and rigorous assessment of bias risk, avoiding the ineffective use of tools not designed for certain types of studies.

The methodological quality and risk of bias of the included studies were assessed independently by four reviewers, all of whom had extensive training and a proven track record in scientific research. The assessment was carried out using the instruments described above. In situations where discrepancies were detected, a joint review was conducted, followed by a critical discussion until consensus was reached. This collaborative process reinforced inter-rater reliability and ensured the consistency of the methodological criteria applied.

### 3.2. Characteristics of the Studies

Table 7 summarizes in detail the main aspects of each study included authors and year of publication, objective, study design, sample characteristics, main results, and effect size or confidence interval when reported. This table provides an organized, comparative overview of the available evidence on interventions and research related to EI and ADHD:

### 3.3. Summary of Results

#### 3.3.1. Characteristics of the Population Analyzed

The 31 studies included constitute a broad and heterogeneous sample in terms of age, gender, and geographical origin. The sample size ranges from 1 to 1388 participants, with a clear predominance of research focused on children and adolescents.

- Most of the studies (15/22) focus on children between the ages of 6 and 12, making this the most represented age group.
- A total of 8 studies include adolescents (12–18 years old).
- Only 5 studies focus on adults, which shows a lower presence of studies in this group.
- Some studies include specific samples such as preschool children (4 years old) or do not report age (2/31).

In terms of sex, there is a clear predominance of males, especially in studies with children and adolescents, where in many cases they exceed 60%. However, some studies present a more balanced distribution or even a greater female presence, especially in samples of adolescents and adults. In 7 studies, sex was not reported.

From a geographical perspective, the studies mainly come from:

- Spain (10/31), Asia (6/31), and Sweden (4/31), followed by other countries such as:
- North America (3/31), Latin America (2/31), Australia (2/31), Italy, France, Romania, Egypt, and Israel (1/31 each).

Geographic diversity contributes to a multicultural view of the relationship between ADHD and emotional intelligence.

In relation to comorbidities, studies frequently identify the presence of: anxiety, depression, anger or low emotional tolerance, behavioral problems, insecure attachment styles, externalizing behaviors, callous–unemotional (CU) traits, and, in some cases, borderline intellectual disability.

These comorbidities enrich the understanding of the emotional profiles associated with ADHD.

Furthermore, with regard to the assessment of risk of bias, it should be noted that, when interpreting the results, the controlled trials analyzed using the ROB-2 (2/31) generally presented a low risk of bias, while the quasi-experimental studies evaluated using the ROBINS-1 (7/31) mostly showed a high risk of bias. In contrast, most of the observational studies assessed using the Newcastle–Ottawa scale (22/31) showed moderate to high methodological quality.

#### 3.3.2. Methodological Analysis

From a methodological perspective, the studies reviewed on EI in populations with ADHD show a marked predominance of quantitative and observational designs, both cross-sectional and longitudinal. A significant portion of the research adopts a cross-sectional approach, which allows for the identification of associations between ADHD and emotional variables, although with the inherent limitation of not being able to establish causal relationships. At the same time, there are longitudinal and quasi-experimental studies, especially in the field of interventions focused on EI training. This type of design offers greater capacity to analyze the evolution of emotional competencies and assess the effectiveness of the programs applied.

Likewise, studies incorporating control groups are relatively common, enabling comparisons between individuals with ADHD and typically developing populations, thereby enriching the interpretation of emotional and socio-adaptive differences. Most studies use standardized instruments to assess EI and emotional regulation, including validated questionnaires, psychometric batteries, and procedures for observing socio-emotional behavior.

It is important to note that the compilation of effect sizes in most studies (*n* = 21) enhances methodological rigor, allowing for the quantification of the impact of interventions. The effect sizes reported in the literature range from small to very large (r ≈ 0.14–0.78), suggesting differences and relationships in executive function and emotional regulation among individuals with ADHD. This consistent presentation of effect sizes strengthens the evidence base, supports the comparability of results across studies, and improves the interpretability of findings in terms of practical implications, beyond statistical significance.

#### 3.3.3. Main Lines of Research Identified

With regard to the lines of research identified, three main approaches can be distinguished. First, a substantial portion of the studies analyze the relationship between ADHD and emotional dysregulation, examining how variables such as affective lability, emotional reactivity, and self-regulation capacity affect the core symptoms of the disorder and social, academic, and behavioral functioning. Second, several studies directly compare EI levels between people with ADHD and control groups, assessing dimensions such as emotional perception and expression, emotion management, adaptability, empathy, and mood. Finally, there is growing interest in educational and therapeutic interventions aimed at developing EI. In this area, studies examine the extent to which specific pedagogical strategies and programs contribute to improving self-control, emotional regulation, and social–emotional skills in children and adolescents with ADHD, demonstrating their positive impact on reducing symptoms and improving overall functioning.

#### 3.3.4. Findings on Emotional Intelligence in ADHD

The analysis of the literature shows a consistent association between ADHD and lower levels of EI, especially in children and adolescents. In general terms, studies agree that people with ADHD have greater difficulties in skills such as emotional management, stress management, adaptability, and interpersonal skills. Recurrently, assessments carried out by external observers (parents, teachers, or other professionals) tend to show lower EI scores than self-reported ones, highlighting possible deficits in emotional perception or expression. Research such as that by Bayarsaikhan et al. [38] and Barahona & Alegre [54] particularly highlights difficulties in the intrapersonal and interpersonal dimensions, including emotional recognition and expression, impulse control, and adaptation to social contexts.

In line with this, studies such as those by Alacha et al. [34] and Jaisle et al. [42] show that emotional dysregulation (a central dimension of EI) plays a mediating role between ADHD symptoms and the onset of emotional and behavioral problems. This mediation suggests that a reduced level of EI can intensify the effects of ADHD, increasing the likelihood of anxiety, depression, aggressive behaviors, and difficulties in social interaction. From this perspective, strengthening EI is presented as a priority strategy for improving the emotional and social well-being of this population.

In terms of intervention, various studies describe positive results associated with specific programs aimed at developing EI. Fontana Abad [33] shows that structured training in emotional skills applied to adolescents with ADHD is associated with significant improvements in EI, self-esteem, and symptom reduction. Complementarily, Villafuerte and Zambrano [32] document how breathing, relaxation, and music therapy techniques promote emotional self-control in young children with ADHD, even in case study designs.

However, the relationship between ADHD and EI in the adult population is more heterogeneous. Quintero et al. [52] finds that some adults with ADHD do not necessarily show significant impairment in EI, pointing to the modulating role of variables such as comorbidity, interventions received, or the acquisition of compensatory strategies. Similarly, Vera García [53] identifies that, although adults with ADHD tend to show lower levels of EI, this is directly associated with quality of life, with hyperactivity/impulsivity being a more relevant predictor than inattention.

Finally, various studies show that difficulties in EI are closely associated with problems in social interaction and everyday functioning. Piltz [40] points out that limited emotional recognition and low socio-emotional competence are related to greater difficulties in interpersonal relationships and poorer school and social adjustment in childhood. Overall, the findings reviewed agree on the central role of EI in understanding ADHD and designing effective interventions. Thus, deficits in EI can exacerbate the symptoms of the disorder and hinder social adaptation, while strengthening EI is an essential component in improving the prognosis, emotional well-being, and quality of life of people with ADHD from the early stages of development.

## 4. Discussion

The results of this review show a consistent, bidirectional relationship between EI and ADHD, demonstrating that emotional difficulties are a central dimension of the clinical profile of the disorder. The reviewed literature indicates that children and adolescents with ADHD have significant deficits in fundamental components of EI, such as emotional regulation, recognition and understanding of their own and others’ emotions, empathy, and stress management [39]. These emotional limitations are directly linked to school adjustment, self-esteem, interpersonal relationships, and the emergence of comorbidities [56]. In line with previous studies, the findings suggest that emotional dysregulation should not be understood as a secondary consequence of the core symptoms of ADHD, but as an element intrinsically related to its clinical expression. Research such as that by Alacha et al. [34] and Jaisle et al. [42] suggests that such dysregulation may mediate the onset of broader problems, such as anxiety, depression, or disruptive behaviors, reinforcing the need for a therapeutic approach that includes specific strategies to strengthen emotional competencies.

Difficulties in IE contribute to maintaining and aggravating the social problems associated with ADHD. The reduced ability to accurately interpret the emotions of others, combined with reduced emotional control, can lead to persistent interpersonal challenges, social rejection, and isolation, especially in the school environment. This dynamic can feed back into the symptoms of the disorder and negatively impact the child’s psychological well-being, creating a vicious cycle that is difficult to break without early and specific interventions [57]. Evidence from studies applying emotional training programs shows particularly promising results. Interventions designed by Fontana Abad [33] and Villafuerte & Zambrano [32] indicate that strengthening emotional competencies not only increases EI levels but also reduces ADHD symptoms, especially in areas related to self-control, impulsivity, and behavioral regulation. These results support the idea that EI is a capacity that can be developed through structured programs, even in populations with neurodevelopmental disorders.

However, despite the general consensus on the presence of emotional deficits in ADHD, the review also highlights significant discrepancies in the literature. Some of these arise when comparing studies focused on different developmental stages. While research on children and adolescents, such as that by Barahona & Alegre [54] and Bayarsaikhan et al. [38], identifies clear deficits in intrapersonal and interpersonal dimensions, studies on adults (such as those by Quintero et al. [52] and Vera García [53]) do not necessarily find lower EI in this population. This divergence may be related to factors such as compensatory strategies developed with age, the impact of life experiences, or the effect of early interventions. Similarly, there are discrepancies in relation to emotional recognition: Piltz [40] documents specific difficulties in identifying emotions such as fear, sadness, or surprise in children with ADHD, while Lasmono et al. [46] find no significant differences in either empathy or general EI. These inconsistencies could be due to methodological differences, variability in assessment instruments, or heterogeneous clinical profiles. In addition, studies such as Fantozzi et al. [41] incorporate additional variables, such as insensitive and non-emotional traits, which can coexist with ADHD and modulate emotional expression, adding a level of complexity that is not always present in designs focused exclusively on EI. These divergences do not invalidate the main body of evidence, but they underscore the need for more precise analyses that consider the influence of developmental, clinical, and contextual factors.

Given these limitations, it is important to consider the methodological quality of the studies as a key factor when interpreting the findings. Assessments of bias measures revealed notable differences depending on study design, identifying higher levels of bias in intervention studies compared to observational studies. This variability explains the heterogeneity of the results reported in the literature, particularly regarding the effectiveness of EI interventions and the magnitude of emotional deficits associated with ADHD. Therefore, the robustness of these conclusions should be interpreted with caution, taking into account the methodological limitations identified in the studies.

A more detailed analysis of the risk of bias across different study designs further qualifies these findings. The results of the assessment reveal considerable heterogeneity depending on the design. Most of the non-randomized intervention studies analyzed using ROBINS-I were at high risk of bias, primarily due to confounding factors, participant selection, and deviations from the planned interventions. This limitation weakens the strength and certainty of the conclusions regarding the impact of EI-based interventions, as causal relationships must be interpreted with caution. In contrast, randomized controlled trials assessed using RoB 2 generally presented a lower risk of bias, although their number was limited, which affects the overall strength of the high-quality evidence. Observational studies, most of which are rated as moderate to high quality according to the Newcastle–Ottawa Scale, provide consistent evidence regarding the associations between ADHD and emotional variables; however, their design does not allow for robust causal conclusions. Taken together, these findings suggest that, although the available evidence points to a beneficial role for EI interventions, the certainty of the evidence is compromised by various methodological biases. This highlights the need for more rigorous randomized controlled trials to strengthen the evidence on the efficacy of these interventions.

The review also identified detected potential risks of bias in several dimensions. Selection bias was reduced through a comprehensive search strategy in six major databases and the consistent application of inclusion and exclusion criteria using Covidence software, which allowed for independent reviews. Publication bias was mitigated by including articles in both Spanish and English and by referring to key studies. With regard to information bias, variability was identified in the tools used to assess EI: some studies used self-reports, while others relied on external observers such as parents or teachers, which may affect the objectivity of the results. This aspect was carefully assessed during the interpretation. Finally, the studies were reviewed to ensure that they adequately reported the results defined in their objectives, considering the possible presence of reporting bias. The identification of these methodological limitations allows the findings to be contextualized and their actual scope to be assessed.

However, it should be noted that the exclusion of the term “emotional regulation” has led to the omission of potentially relevant publications due to the conceptual overlap with EI in the literature on ADHD. The decision was justified by the delimitation criteria, but it is possible that certain interventions aimed at emotional skills have been developed under the terminology of emotional regulation.

Likewise, the use of criteria open access as an inclusion criterion may have introduced a selection bias based on accessibility rather than on the scientific quality or relevance of the studies. Potentially relevant research may not have been included due to access restrictions derived from the publication model (subscription or hybrid format). This circumstance could affect the representativeness of the evidence collected and limit the scope of the findings. Future reviews should consider alternative access strategies, such as direct requests to authors or the use of interlibrary services, in order to minimize this potential bias.

Based on the above, several future lines of research and intervention can also be identified. Longitudinal studies are particularly necessary to understand how emotional competencies evolve from childhood to adulthood, identifying whether emotional deficits persist, worsen, or can be compensated for with maturation. Likewise, the literature points to the importance of designing integrated interventions that combine emotional training with cognitive and behavioral techniques, incorporating EI as a central axis to increase therapeutic efficacy. In the educational field, it is proposed that emotional education be systematically included in school programs, both to prevent difficulties and to support diagnosed students. The need to personalize interventions, taking into account variables such as gender, ADHD subtype, or the presence of comorbidities, is also highlighted. The use of new technologies (such as mobile applications, therapeutic video games, or virtual reality) could facilitate more attractive and effective emotional training. Finally, the importance of training parents and teachers as agents of emotional support in the child’s everyday environment is highlighted, thus reinforcing more balanced and secure development.

Overall, the results of this review highlight the need to explicitly integrate emotional intelligence into the approach to ADHD. Moving toward more comprehensive intervention models that go beyond a fragmented view focused solely on symptoms will enable the development of more effective, tailored, and humanized strategies for the clinical and educational support of people with ADHD.

## 5. Conclusions

This review shows that EI is a key component in understanding the adaptive functioning of people with ADHD. Although previous research has tended to focus on cognitive and behavioral symptoms, the available evidence confirms that emotional difficulties are part of the functional core of the disorder and contribute significantly to its clinical variability. The alterations observed in emotional regulation, affective recognition, empathy, or stress management are directly related to quality of life, social adjustment, and academic performance.

The studies reviewed suggest that EI not only reflects the consequences of ADHD, but can also influence the intensity and persistence of its manifestations. This integrative perspective provides a better understanding of why some profiles show greater emotional vulnerability and why socio-adaptive development may be compromised even in the absence of severe behavioral symptoms. In addition, the review highlights the existence of heterogeneous developmental trajectories, reinforcing the need to consider EI as a factor that modulates the course of the disorder.

In the field of intervention, programs aimed at developing emotional skills show positive results and represent a promising way to complement traditional approaches. Although the benefits observed are consistent, most studies offer limited follow-up and use instruments that are not always adapted to the population with ADHD, highlighting the need to improve assessment tools and strengthen longitudinal evidence. Even so, the findings indicate that training EI can promote emotional autonomy, self-control, and interpersonal functioning, contributing significantly to overall well-being.

In summary, integrating EI into ADHD assessment and intervention models is a fundamental strategy for advancing toward a more complete and accurate understanding of the disorder. Future studies should consider the diversity of clinical profiles, the role of comorbidities, developmental differences, and family and school contexts in order to design more tailored and personalized interventions. Strengthening EI not only improves symptom management but also offers a solid pathway to promoting more balanced development and a higher quality of life at all stages of the life cycle.

## Figures and Tables

**Table 1:** PICOS strategy descriptors.

Components	Description
“P”	Population: People with ADHD
“I”	Constructs of Interest: Interventions aimed at the development of EI
“C”	Comparison: Not included, as no comparative analyses were performed.
“O”	Results/Outcomes: Characteristics of interventions, effect size, and effectiveness
“S”	Designs: Empirical studies using qualitative, quantitative, or mixed methodologies

**Table 2:** Search equations.

Search Equations	Databases	Results
(ADHD OR Attention Deficit Hyperactivity Disorder) AND (Emotional Intelligence OR Social Emotional Learning OR EQ) AND (Intervention OR Treatment OR Program OR Therapy) NOT (“Emotional Regulation”).	PubMed	149
(ADHD OR Attention Deficit Hyperactivity Disorder) AND (Emotional Intelligence OR Social Emotional Learning OR EQ) AND (Intervention OR Treatment OR Program OR Therapy) NOT (“Emotional Regulation”).	Web Of Science	185
(ADHD OR Attention Deficit Hyperactivity Disorder) AND (Emotional Intelligence OR Social Emotional Learning OR EQ) AND (Intervention OR Treatment OR Program OR Therapy) NOT (Emotional Regulation).	Scopus	376
(ADHD OR Attention Deficit Hyperactivity Disorder) AND (Emotional Intelligence OR Social Emotional Learning OR EQ)) AND (Intervention OR Treatment OR Program OR Therapy) NOT (“Emotional Regulation”).	Dialnet	132
(ADHD OR Attention Deficit Hyperactivity Disorder) AND (Emotional Intelligence OR Social Emotional Learning OR EQ) AND (Intervention OR Treatment OR Program OR Therapy) NOT (“Emotional Regulation”).	SpringerLink	1331
(ADHD OR Attention Deficit Hyperactivity Disorder) AND (Emotional Intelligence OR Social Emotional Learning OR EQ) AND (Intervention OR Treatment OR Program OR Therapy) NOT (“Emotional Regulation”).	ERIC	163

**Table 3:** Inclusion and exclusion criteria.

Inclusion Criteria	Exclusion Criteria
Empirical research or interventions examining EI in individuals with ADHD	Interventions that do not focus primarily on IE
Studies involving children, adolescents, adults, or family members of individuals with ADHD	Studies focusing on disorders other than ADHD
Articles published in any geographical or linguistic context (Spanish or English)	Sample not aligned with the study objective
Studies with full-text available (open access)	Articles in languages other than Spanish or English

**Table 4:** RoB2 quality assessment.

Study	D1	D2	D3	D4	D5	Risk
Wong et al., (2024) [25]	Some concerns	Some concerns	Some concerns	Low risk	Low risk	Some concerns
Choi & Lee (2015) [26]	Some concerns	Some concerns	Low risk	High risk	Some concerns	High risk

**Table 5:** ROBINS-I quality assessment.

Study	D1	D2	D3	D4	D5	D6	D7	Risk
Cabello-Sanz et al., (2025) [27]	Serious	Serious	Low	Moderate	Low	Moderate	Moderate	Serious
Su et al., (2024) [28]	Serious	Serious	Low	Moderate	Moderate	Serious	Moderate	Serious
Takim et al., (2024) [29]	Critic	Moderate	Low	Moderate	Serious	Moderate	Moderate	Serious
Zhang et al., (2024) [30]	Serious	Moderate	Low	Moderate	Low	Moderate	Moderate	Serious
Löytömäki et al., (2023) [31]	Serious	Moderate	Low	Moderate	Low	Moderate	Moderate	Serious
Villafuerte & Zambrano (2023) [32]	Serious	Serious	Moderate	Low	Low	Moderate	Moderate	Serious
Fontana Abad, (2015) [33]	Serious	Serious	Low	Low	Moderate	Low	Moderate	Serious

**Table 6:** Article quality assessment process.

Study	Selection	Comparability	Results	Total	Quality	Bias
Alacha et al., (2024) [34]	4	2	3	9	HQ	Low
Hulsbosch et al., (2024) [35]	4.5	1.5	2.5	8.5	HQ	Low
Martin et al., (2024) [36]	4	1	3	8	HQ	Low
Rosenblum et al., (2024) [37]	4	2	3	9	HQ	Low
Bayarsaikha et al., (2023) [38]	4	1.5	2	7.5	QM	Medium
Ben Turkia et al., (2023) [39]	3.5	1	2.5	7	QM	Medium
Piltz, (2023) [40]	4	1.5	2.5	8	HQ	Low
Fantozzi et al., (2022) [41]	4	2	2.5	8.5	HQ	Low
Jaisle et al., (2022) [42]	4.5	2	2.5	9	HQ	Low
Navarro-Noguera &Herrera-Gutiérrez, (2022) [43]	3.5	0	2.5	6	QM	Medium
Tallberg et al., (2022) [44]	4.5	2	2.5	9	HQ	Low
Battistutta et al., (2021) [45]	4.5	2	2.5	9	HQ	Low
Lasmono et al., (2021) [46]	4.5	1.5	2.5	8.5	HQ	Low
García López & Leal Baeza (2020) [47]	3.5	1	1.5	6	QM	Medium
Predescu et al., (2020) [48]	4	1.5	2.5	8	HQ	Low
Eyuboglu & Eyuboglu (2020) [49]	4	2	2.5	8.5	HQ	Low
Amin Yazdi et al., (2018) [50]	3	1.5	2.5	7	QM	Medium
Abo Elella et al., (2017) [51]	4	1.5	2.5	8	HQ	Low
Quintero et al., (2017) [52]	3.5	1.5	2	7	QM	Medium
Vera Garcia, (2017) [53]	4.5	2	2.5	9	HQ	Low
Barahona & Alegre (2016) [54]	3.5	1	2.5	7	QM	Medium
Kristensen et al., (2014) [55]	4	2	3	9	HQ	Low

**Table 7:** Summary of studies.

Authors Year	Objectives	Design	Sample	Results	Effect Size/Confidence Interval
Cabello-Sanz et al., (2025) [27]	Analyze the effectiveness of the PEECE (Cooking Emotions Emotional Education Program) project on academic performance in primary school students with ADHD	Quasi-experimental Diagnostic tools: NR	Participants: 108 Age: 8–12 years old Gender: 75.76% male and 24.24% female Country: Spain Target population: Children with ADHD. Frequency: 1 session per week for 3 months Setting: Clinical	The analysis showed that after the intervention, the number of students in the experimental group with higher academic performance and greater emotional skills increased, suggesting a positive association between EI and academic performance in children with ADHD.	Effect size: NR CI: NR
Alacha et al., (2024) [34]	Analyze the role of emotional dysregulation (as a component of EI) in the relationship between ADHD symptoms and emotional and behavioral problems	Quantitative, longitudinal Diagnostic tools: DSM-IV	Participants: 215 Age: 8–12 years old Gender: 67.6% male and 32.4% female Country: Spain Target population: Children with and without ADHD Frequency: NR Setting: Community-based	Emotional reactivity predicts the future severity of symptoms of inattention, hyperactivity/impulsivity, and total ADHD, beyond initial severity. On the other hand, emotional lability does not predict symptom severity, but negative affect variability does predict hyperactivity/impulsivity and total ADHD. Initial ADHD severity also did not predict subsequent changes in emotional regulation.	Effect size: r ≈ 0.35–0.40 CI: NR
Hulsbosch et al., (2024) [35]	Analyze the relationship between emotional profile and externalized behavior in children with a clinical diagnosis of ADHD	Cross-sectional, observational Diagnostic tools: DSM-5, K-SADS, Clinical Assessment	Participants: 36 Age: 6–12 years old Gender: 64.15% male and 35.85% female Country: Sweden Target population: Children with and without ADHD Frequency: NR Setting: Laboratory (experimental)	A significant relationship was found between a more disturbed emotional profile (greater emotionality and less emotional control) and greater behavioral disturbance (externalized behaviors such as aggression, hyperactivity, or attention problems).	Effect size: r ≈ 0.20–0.32 CI: NR
Martin et al., (2024) [36]	Analyze differences in social motivation capacity (interpersonal EI) in children and adolescents with ADHD and its relationship with emotional regulation and EI	Quantitative, cross-sectional Diagnostic tools: Parental self-report, DAWBA symptoms	Participants: 204 Age: 5–8 years old Gender: 59% male and 41% female Country: Australia Target population: Parents of children and adolescents with ADHD Frequency: N/A Setting: Online/Community-based	Children and adolescents with ADHD show significantly lower social motivation and interpersonal EI than typically developing adolescents. The degree of emotional regulation significantly predicts lower social motivation in children and adolescents with ADHD.	Effect size: r ≈ 0.81 CI: 95%
Rosenblum et al., (2024) [37]	Analyze how temporal disorganization predicts emotional responses in adults with ADHD	Quantitative cross-sectional Diagnostic tools: DSM-V-TR	Participants: 290 Age: 20–50 years old Gender: 34.20% male and 65.80% female Country: Israel Target population: Adults with ADHD Frequency: NR Setting: Clinical	Difficulties in executive functions and time management are significantly associated with negative emotional responses (frustration, stress, low motivation). Poor time management mediates the relationship between cognitive deficits and emotional consequences, being more pronounced in adults with ADHD.	Effect size: r ≈ 0.56–0.71 CI: 95%
Su et al., (2024) [28]	Analyze the impact of an AI-based program for the development of IE in students with ADHD	Quasi-experimental Diagnostic tools: Psychoeducational assessment, DSM-5 criteria	Participants: 22 Age: NR Gender: NR Country: Taiwan Target population: Children with ADHD Frequency: One session per week for 3 months Setting: School	The sample from 5th and 6th grade elementary school showed improvements in five dimensions of EI: self-awareness, self-control, social awareness, relationship skills, and responsible decision-making.	Effect size: r ≈ 0.48–0.57 CI: NR
Takım et al., (2024) [29]	Analyze changes in IE capacity in the population with ADHD following the development of a drug intervention	Quasi-experimental Diagnostic tools: Structured Clinical Interview (SCID-5-CV), DSM-5 criteria	Participants: 50 Age: 22.20 years old Gender: 54% male and 46% female Country: Turkey Target population: Adults with ADHD Frequency: Every 3 months Setting: Clinical	Following the intervention, improvements were observed in both EI capacity and ADHD. The sample showed a significant reduction in symptoms associated with ADHD and narcissism and strengthened empathy. The program had a positive impact on social and interpersonal functioning.	Effect size: NR CI: NR
Wong et al., (2024) [25]	Examine the effectiveness of a virtual reality-based social skills training intervention to improve emotional and social competencies	Randomized controlled Diagnostic tools: Preliminary diagnosis by healthcare professionals	Participants: 90 Age: 6–12 years old Gender: 77% male and 23% female Country: Hong Kong Target population: Children with ADHD Frequency: 12 sessions spread over 3 months Setting: Community-based	The population that participated in the program showed significant improvements in their social skills, self-control, initiative, and emotional control. The intervention developed showed high acceptability and adherence.	Effect size: Self-control: r ≈ 0.21 Initiative: r ≈ 0.12 Emotional control: r ≈ 0.20 IC: 95%
Zhang et al., (2024) [30]	Analyze the effectiveness of an AI program to improve the educational outcomes of children with ADHD and reduce their limitations.	Quasi-experimental Diagnostic tools: Prior diagnosis by physicians	Participants: 16 Age: 7–12 years old Gender: 62.5% male and 37.5% female Country: China Target population: Children with ADHD Frequency: Every 2 weeks, with 20 min sessions Setting: Clinical	The sample showed a significant reduction in ADHD symptoms. Improvements were also observed in some skills associated with EI, such as greater emotional expression, emotional self-regulation, concentration, and motivation, less frustration, and greater involvement in tasks.	Effect size: NR CI: NR
Bayarsaikhan et al., (2023) [38]	Compare IE between children with ADHD and a control group after the program	Observational, cross-sectional Diagnostic tools: DSM-IV	Participants: 30 Age: 7–12 years old Gender: 24 boys and 6 girls Country: Mongolia Target population: Children with ADHD Frequency: NR Setting: School	Children with ADHD had lower levels of total EI and in the intrapersonal, interpersonal, adaptability, stress management, and general mood dimensions compared to the control group. On the other hand, differences were observed between ADHD subtypes in the intrapersonal and adaptability dimensions.	Effect size: r ≈ 0.37 CI: NR
Ben Turkia et al., (2023) [39]	Compare emotional and social functioning in children with and without ADHD	Cross-sectional, comparative Diagnostic tools: NR	Participants: 60 Age: NR Gender: NR Country: France Target population: Children and adolescents with ADHD Frequency: NR Setting: Clinical	Children and adolescents with ADHD have a significantly lower emotional quotient than the control group. Lower scores are observed in interpersonal skills, adaptability, general mood, and positive impression, indicating greater difficulties in EI compared to the population without ADHD.	Effect size: r ≈ 0.41 for total EI; r ≈ 0.52 for specific facets CI: NR
Löytömäki et al., (2023) [31]	Examine the impact of the “Emotion Detectives” program on the dimensions of EI (emotional discrimination, social–emotional capacity) in children with ADHD	Quasi-experimental Diagnostic tools: Preliminary diagnosis based on a parent-reported clinical assessment	Participants: 30 Age: 8.02 years Gender: 6 male and 24 female Country: Finland Target population: Children with neurodevelopmental disorders (ADHD) Frequency: 1 session per week for 8 weeks Setting: Mixed (home, school, clinical)	After two months of intervention, improvements were observed in emotional discrimination. During follow-up, it was observed that the benefits were maintained for one month. Parents reported that their children had developed their emotional recognition skills. Improvements in emotional discrimination of tone of voice predicted part of the behavioral improvement.	Effect size: r ≈ 0.32–0.50 CI: NR
Piltz (2023) [40]	Analyze the difficulties in social interaction experienced by children with ADHD and their relationship with emotional aspects such as EI	Comparative observational Diagnostic tools: DSM-IV, CIE-10	Participants: 60 Age: 8–12 years old Gender: 50% male and 50% female Country: Sweden Target population: Children and adolescents with ADHD Frequency: NR Setting: Clinical	Children and adolescents with ADHD show differences in socioemotional expression during social interaction, displaying greater use of gestures with emotional content compared to the group without ADHD. These findings suggest particularities in emotional expression associated with ADHD.	Effect size: r ≈ 0.31 CI: 95%
Villafuerte & Zambrano (2023) [32]	Provide tools so that children with ADHD can apply self-control through EI	Quasi-experimental Diagnostic tools: Clinical diagnosis, Conners Behavior Questionnaire	Participants: 1 Age: 4 years old Gender: 100% male Country: Ecuador Target population: Children with ADHD Frequency: Twice a week for 3 months Setting: School	The study found significant improvements in EI indicators and emotional self-control following the intervention applied during the study.	Effect size: NR CI: NR
Fantozzi et al., (2022) [41]	Investigate the relationship between insensitive and unemotional traits and intelligence in children with externalizing behavior problems	Quantitative, cross-sectional, correlational Diagnostic tools: Prior clinical diagnosis	Participants: 98 Age: 9.03 years Gender: 84.6% male and 15.4% female Country: Italy Target population: Children with ADHD Frequency: NR Setting: Clinical	Callous–unemotional traits are negatively associated with EI (emotional understanding), even after controlling for age, gender, and externalizing problems. No significant associations with other cognitive areas or interaction with the severity of behavioral problems were observed.	Effect size: r ≈ 0.34 CI: 95%
Jaisle et al., (2022) [42]	Analyze how emotional dysregulation influences the relationship between ADHD symptoms and emotional and behavioral problems in childhood and explore the mediating role of EI	Cross-sectional, correlational Diagnostic tools: DSM-V	Participants: 215 Age: 8–12 years old Gender: 62% male and 38% female Country: USA Target population: Children with and without ADHD Frequency: NR Setting: Clinical	Symptoms of inattention in ADHD are directly associated with greater social impairment. Symptoms of hyperactivity/impulsivity indirectly predict social impairment through emotional dysregulation. Emotional dysregulation is negatively associated with social competence, even when controlling for symptoms of ADHD and ASD.	Effect size: r ≈ −0.23 CI: NR
Navarro-Noguera & Herrera-Gutiérrez, (2022) [43]	Describe the cognitive profile of adolescents with ADHD and its educational implications	Cross-sectional description Diagnostic tools: DSM-V	Participants: 127 Age: 12–18 years old Gender: 81.89% male and 18.11% female Country: Spain Target population: Adolescents with ADHD Frequency: NR Setting: Clinical	Adolescents with ADHD have average scores in general intelligence. They perform better in nonverbal skills (matrix reasoning) than in verbal skills (vocabulary), suggesting a strength in nonverbal reasoning and greater relative difficulties in verbal processing. Their IQ is within the average range, tending toward the high-normal range.	Effect size: NR CI: 95%
Tallberg et al., (2022) [44]	Examine the emotional symptoms of young people with ADHD following an intervention process	Longitudinal observational Diagnostic tools: DSM-IV	Participants: 137 Age: 12.4 years Gender: 70.07% male and 29.93% female Country: Sweden Target population: Children and adolescents with ADHD Frequency: NR Setting: Clinical	Young people with ADHD have greater difficulties in their emotional abilities compared to the control group. These difficulties are significantly associated with problems in emotional regulation, especially in emotional control and planning/organization, variables related to EI.	Effect sizes: r ≈ 0.30 CI: 95%
Battistutta et al., (2021) [45]	Examine the role of emotional dysregulation as a mediator between ADHD and children’s daily functioning	Quantitative, cross-sectional, mediational Diagnostic tools: DSM-V	Participants: 183 Age: 7–11 years old Gender: 62% male and 38% female Country: USA Target population: Children and adolescents with ADHD Frequency: NR Setting: Clinical	Working memory deficits are associated with increased symptoms of anxiety and depression through inattention or hyperactivity and difficulties with emotional regulation. Hyperactivity and emotional regulation mediate the relationship between attentional control and inhibition with internalizing symptoms, while inattention does not show a consistent mediating role.	Effect size: r ≈ 0.27 (anxiety); r ≈ 0.24 (depression) CI: 95%
Lasmono et al., (2021) [46]	Analyze levels of empathy and systematization in primary school children with and without ADHD	Cross-sectional, comparative, and observational Diagnostic tools: ICD-10 and the Conners Scale	Participants: 122 Age: 7 to 12 years old Country: Indonesia Gender: 63.11% male and 36.89% female Target population: Parents of children with ADHD Frequency: NR Setting: Clinical	Children with ADHD showed lower empathy scores compared to the group without ADHD, regardless of gender. In the systematization, significant differences were observed only in girls, with lower scores in the ADHD group.	Effect size: r ≈ 0.14–0.54 IC: NR
García López & Leal Baeza (2020) [47]	Analyze the role of EI and self-determined motivation in students with ADHD, as well as their influence on satisfaction and frustration	Descriptive and experiential Educational innovation project and family support groups Diagnostic tools: DSM-V	Participants: 1143 Age: 10 years old Gender: NR Country: Spain Target population: Children with ADHD Frequency: NR Setting: Educational	Higher EI is related to greater self-determined motivation, which predicts greater satisfaction of basic psychological needs and less frustration. Working on EI can improve school and emotional adjustment in children with ADHD.	Effect size: NR CI: NR
Predescu et al., (2020) [48]	Analyze the relationship between executive functions, emotional regulation, and emotional and behavioral problems in children with ADHD	Quantitative, cross-sectional, comparative Diagnostic tools: CIE-10	Participants: 85 Age: 6 to 11 years old Gender: 47.61% boys and 52.39% girls Country: Romania Target population: Children with ADHD Frequency: NR Setting: Clinical	Children with ADHD show greater use of maladaptive emotional regulation strategies and higher levels of anxiety and behavioral problems than the typically developing group. Adaptive emotional regulation is positively associated with executive functions, while poorer control is related to more ADHD symptoms and emotional problems.	Effect size: r = 0.24–0.36 CI: NR
Eyuboglu & Eyuboglu (2020) [49]	Examine the relationship between emotional regulation and attachment styles in adolescents with ADHD who have not received prior treatment	Clínic Diagnostic tools: DSM-V-TR	Participants: 99 Age: 12 to 17 years old Gender: 80% male and 20% female Country: Turkey Target population: Adolescents with ADHD Frequency: NR Setting: Clinical	Adolescents with untreated ADHD show greater difficulties in emotional regulation compared to the control group. It is possible that insecure attachment styles are more prevalent in the ADHD group.	Effect size: r ≈ 0.33–0.45 CI: NR
Amin Yazdi et al., (2018) [50]	Compare emotional intelligence and cognitive flexibility in children with and without ADHD, and analyze whether EI predicts cognitive flexibility	Cross-sectional, comparative, correlational Diagnostic tools: Previous clinical diagnosis	Participants: 50 Age: 8–14 years old Gender: NR Country: Iran Target population: Children with ADHD Frequency: NR Setting: Mixed (educational, home, clinical)	Children with ADHD showed significantly lower scores in EI and cognitive flexibility. EI predicts 27% of the variability in cognitive flexibility. The importance of training EI to improve emotional and cognitive functioning in children with ADHD is highlighted.	Effect size: r = 0.69 CI: NR
Abo Elella et al., (2017) [51]	Examine the relationship between ADHD subtypes and symptoms and TEI in Egyptian children	Cross-sectional, comparative, correlational Diagnostic tools: DSM-IV	Participants: 75 Age: 8–12 years old Gender: NR Country: Egypt Target population: Children with ADHD Frequency: NR Setting: Clinical	Children with ADHD have significantly lower scores on global EI and most of its facets compared to controls, except for adaptability. Mixed and hyperactive subtypes show greater emotional impairment than the inattentive subtype.	Effect size: r ≈ 0.37 CI: NR
Quintero et al., (2017) [52]	Analyzes the level of development of EI as a skill in adults with ADHD, examining the impact of psychiatric comorbidity and a previous diagnosis of ADHD in childhood or adolescence	Cross-sectional, comparative, and observational Diagnostic tools: DSM-IV-TR	Participants: 116 Age: 38.29 years old. Gender: 45.2% male and 54.8% female. Country: Spain Target population: Adults with ADHD Frequency: NR Setting: Clinical	Significant differences were found between groups in overall EI and in the dimensions “Use of emotions to facilitate thinking” and “Emotional understanding.” Adults with ADHD and comorbidity without prior diagnosis had significantly lower levels of overall EI compared to the control group. No significant differences were observed between groups in the dimensions of perception and management of emotions. The severity of ADHD symptoms in childhood or adulthood did not correlate significantly with the current level of emotional intelligence.	Effect size: NR CI: NR
Vera Garcia (2017) [53]	Evaluate IE levels in adults with ADHD and their relationship with quality of life (QoL)	Cross-cutting Diagnostic tools: DSM-V	Participants: 116 Age: 38 years old Gender: NR Country: Spain Target population: Adults with ADHD Frequency: NR Setting: Clinical	Adults with ADHD have lower EI (especially in perception and emotional facilitation) and poorer quality of life than the control group. A positive and significant relationship was found between EI and QoL. Hyperactivity/impulsivity (but not inattention) was associated with lower EI. Women showed higher EI than men, except in cases with comorbidity without prior diagnosis. IQ correlated weakly and positively with EI.	Effect size: NR CI: NR
Barahona & Alegre (2016) [54]	Compare the executive function abilities of adolescents diagnosed with ADHD and adolescents without ADHD	Quantitative, non-experimental, descriptive-comparative, cross-sectional study Diagnostic tools: Prior clinical diagnosis	Participants: 236 Age: 14.07 years old Gender: 79.5% male and 20.5% female Country: Peru Target population: Children and adolescents with ADHD Frequency: NR Setting: Educational	Statistically significant differences between the groups were found only in intrapersonal ability and positive impression, with higher scores in the ADHD group. No significant differences were found in the total emotional quotient, interpersonal dimensions, adaptability, stress management, or general mood.	Effect size: r = −0.15 (intrapersonal), r = −0.14 (positive impressions) CI: 95%
Choi & Lee (2015) [26]	Analyze the impact of emotional management training versus social skills training on improving emotional recognition, expression, and regulation in children with ADHD	Randomized controlled Diagnostic tools: DSM-IV	Participants: 72 Age: 9–13 years old Gender: 44% male and 56% female Country: South Korea Target population: Children with ADHD Frequency: 16 sessions, once a week Setting: Clinical	The sample showed significant improvements in emotional recognition and emotional expression. It also showed greater improvements in social initiative and empathy/cooperation. The findings justify that directly intervening in emotional identification and expression is more effective than focusing solely on social skills.	Effect size: NR CI: NR
Fontana Abad (2015) [33]	Analyze the effectiveness of an EI training program for adolescents with ADHD	Quasi-experimental Diagnostic tools: NR	Participants: 40 Age: 10 to 17 years old Gender: NR Country: Spain Target population: Adolescents with ADHD Frequency: N/A Setting: Educational	The experimental group showed significant improvement in EI, self-esteem, and reduction in ADHD symptoms compared to the control group. The effectiveness of the program is confirmed.	Effect size: NR CI: NR
Kristensen et al., (2014) [55]	Examine the relationship between trait EI (TEI) and ADHD symptoms	Cross-sectional, correlational Diagnostic tools: DSM-5	Participants: 1388 Age: 14–17 years old Gender: 44.30% male and 55.69% female Country: Canada Target population: Adolescents with ADHD Frequency: NR Setting: Educational	TEI predicts ADHD symptoms. Stress management and adaptability predict hyperactivity/impulsivity and inattention. The strongest associations are seen in adults.	Effect size: Adolescents: r ≈ 0.51 Adults: r ≈ 0.78 CI: 90%

Notes: ADHD: Attention Deficit Hyperactivity Disorder, AI: Artificial intelligence, CI: Confidence interval, DSM-IV: Diagnostic and Statistical Manual of Mental Disorders, Four Edition; DSM-V: Diagnostic and Statistical Manual of Mental Disorders, Fifth Edition; CIE-10: International Classification of Diseases, 10th Revision; NR: Not reported, TEI: Emotional Intelligence Trait, PEECE: Cooking Emotions Emotional Education Programme, QoL: Quality of life.

## Data Availability

All the data supporting the findings of this study are contained within the manuscript.

## Abbreviations

The following abbreviations are used in this manuscript:

ADHD	Attention Deficit Hyperactivity Disorder
AI	Artificial Intelligence
AEMPS	Spanish Agency of Medicines and Medical Devices
APA	American Psychiatric Association
CI	Confidence Interval
CU	Callous–Unemotional Traits
DSM-5-TR	Diagnostic and Statistical Manual of Mental Disorders
EQ	Emotional Quotient
ERIC	Education Resources Information Center
HQ	High Quality
IE	Emotional Intelligence
ICE	International Classification of Diseases
LQ	Low Quality
NOS	Newcastle–Ottawa Scale
NR	Not Reported
PEECE	Cooking Emotions Emotional Education Programme
PRISMA	Preferred Reporting Items for Systematic Reviews and Meta-Analyses
PROSPERO	International Prospective Register of Systematic Reviews
QoL	Quality of Life
QM	Medium Quality
TEI	Trait Emotional Intelligence
WoS	Web of Science

## Appendix A

**Table A1:** PRISMA Checklist.

Section and Topic	Item #	Checklist Item	Location Where Item is Reported
TITLE			
Title	1	Identify the report as a systematic review.	1
ABSTRACT			
Abstract	2	See the PRISMA 2020 for Abstracts checklist.	1
INTRODUCTION			
Rationale	3	Describe the rationale for the review in the context of existing knowledge.	1
Objectives	4	Provide an explicit statement of the objective(s) or question(s) the review addresses.	3
METHODS			
Eligibility criteria	5	Specify the inclusion and exclusion criteria for the review and how studies were grouped for the syntheses.	5
Information sources	6	Specify all databases, registers, websites, organisations, reference lists and other sources searched or consulted to identify studies. Specify the date when each source was last searched or consulted.	3
Search strategy	7	Present the full search strategies for all databases, registers and websites, including any filters and limits used.	4
Selection process	8	Specify the methods used to decide whether a study met the inclusion criteria of the review, including how many reviewers screened each record and each report retrieved, whether they worked independently, and if applicable, details of automation tools used in the process.	5
Data collection process	9	Specify the methods used to collect data from reports, including how many reviewers collected data from each report, whether they worked independently, any processes for obtaining or confirming data from study investigators, and if applicable, details of automation tools used in the process.	5
Data items	10a	List and define all outcomes for which data were sought. Specify whether all results that were compatible with each outcome domain in each study were sought (e.g., for all measures, time points, analyses), and if not, the methods used to decide which results to collect.	5
	10b	List and define all other variables for which data were sought (e.g., participant and intervention characteristics, funding sources). Describe any assumptions made about any missing or unclear information.	9
Study risk of bias assessment	11	Specify the methods used to assess risk of bias in the included studies, including details of the tool(s) used, how many reviewers assessed each study and whether they worked independently, and if applicable, details of automation tools used in the process.	5
Effect measures	12	Specify for each outcome the effect measure(s) (e.g., risk ratio, mean difference) used in the synthesis or presentation of results.	9
Synthesis methods	13a	Describe the processes used to decide which studies were eligible for each synthesis (e.g., tabulating the study intervention characteristics and comparing against the planned groups for each synthesis (item #5)).	4
	13b	Describe any methods required to prepare the data for presentation or synthesis, such as handling of missing summary statistics, or data conversions.	8
	13c	Describe any methods used to tabulate or visually display results of individual studies and syntheses.	9
	13d	Describe any methods used to synthesize results and provide a rationale for the choice(s). If meta-analysis was performed, describe the model(s), method(s) to identify the presence and extent of statistical heterogeneity, and software package(s) used.	9
	13e	Describe any methods used to explore possible causes of heterogeneity among study results (e.g., subgroup analysis, meta-regression).	NR
	13f	Describe any sensitivity analyses conducted to assess robustness of the synthesized results.	NR
Reporting bias assessment	14	Describe any methods used to assess risk of bias due to missing results in a synthesis (arising from reporting biases).	6
Certainty assessment	15	Describe any methods used to assess certainty (or confidence) in the body of evidence for an outcome.	6
RESULTS			
Study selection	16a	Describe the results of the search and selection process, from the number of records identified in the search to the number of studies included in the review, ideally using a flow diagram.	5
	16b	Cite studies that might appear to meet the inclusion criteria, but which were excluded, and explain why they were excluded.	NR
Study characteristics	17	Cite each included study and present its characteristics.	9
Risk of bias in studies	18	Present assessments of risk of bias for each included study.	6
Results of individual studies	19	For all outcomes, present, for each study: (a) summary statistics for each group (where appropriate) and (b) an effect estimate and its precision (e.g., confidence/credible interval), ideally using structured tables or plots.	NR
Results of syntheses	20a	For each synthesis, briefly summarise the characteristics and risk of bias among contributing studies.	16
	20b	Present results of all statistical syntheses conducted. If meta-analysis was done, present for each the summary estimate and its precision (e.g., confidence/credible interval) and measures of statistical heterogeneity. If comparing groups, describe the direction of the effect.	NR
	20c	Present results of all investigations of possible causes of heterogeneity among study results.	16
	20d	Present results of all sensitivity analyses conducted to assess the robustness of the synthesized results.	NR
Reporting biases	21	Present assessments of risk of bias due to missing results (arising from reporting biases) for each synthesis assessed.	6
Certainty of evidence	22	Present assessments of certainty (or confidence) in the body of evidence for each outcome assessed.	6
DISCUSSION			
Discussion	23a	Provide a general interpretation of the results in the context of other evidence.	18
	23b	Discuss any limitations of the evidence included in the review.	19
	23c	Discuss any limitations of the review processes used.	19
	23d	Discuss implications of the results for practice, policy, and future research.	19
OTHER INFORMATION			
Registration and protocol	24a	Provide registration information for the review, including register name and registration number, or state that the review was not registered.	3
	24b	Indicate where the review protocol can be accessed, or state that a protocol was not prepared.	3
	24c	Describe and explain any amendments to information provided at registration or in the protocol.	NR
Support	25	Describe sources of financial or non-financial support for the review, and the role of the funders or sponsors in the review.	20
Competing interests	26	Declare any competing interests of review authors.	20
Availability of data, code and other materials	27	Report which of the following are publicly available and where they can be found: template data collection forms; data extracted from included studies; data used for all analyses; analytic code; any other materials used in the review.	20
